# Clinical assessment of breastfeeding in preterm infants

**DOI:** 10.1038/s41430-024-01471-3

**Published:** 2024-07-09

**Authors:** Meredith Kinoshita, Martin J. White, Anne Doolan

**Affiliations:** 1 The Coombe Hospital, Dublin, Ireland; 2 Royal College of Surgeons Ireland, Dublin, Ireland; 3 University College Dublin, Dublin, Ireland

**Keywords:** Nutrition, Paediatrics

## Abstract

Breastmilk confers empirical benefits for preterm infants, however direct breastfeeding rates in this population remain low. For preterm infants, it may be useful to assess the volume of breastmilk transferred from mother to baby when breastfeeding, particularly during transition to oral feeding when breastfeeding attrition is high. Establishing breastfeeding in preterm infants is complex and without knowledge of milk intake during breastfeeds there is risk of inaccurate feed supplementation with subsequent effects on growth and nutrition. Here we review the evidence for clinical assessments of breastfeeding in preterm infants including test weighing, use of isotope labelled water and clinical observation tools designed to estimate adequacy of breastfeeds. Test weighing is a validated measurement, however requires rigorous protocols and further investigation in small infants. Use of isotope labelled water is a validated technique but, due to sampling requirements, reflects intake over days and weeks instead of individual feeds. Clinical observation tools assessed in preterm infants, have not been shown to reflect volumes of breastmilk intake. While current methods have limitations, the goal is to identify measurement tools to be used as temporary aids to facilitate transition to direct breastfeeding while minimising risk of inaccurate supplementation.

## Background

Breastmilk is an essential component of care for preterm infants with clear empirical benefits. Feeding with breastmilk reduces the risk of necrotising enterocolitis, late onset sepsis [1, 2], bronchopulmonary dysplasia [3], retinopathy of prematurity, [4] readmission to hospital within the first year of life [5, 6] and is associated with improved neurodevelopmental outcomes [7] and improved cardiac function [8]. Nevertheless, prevalence of direct breastfeeding remains low in this population [9]. Preterm birth is a risk factor for delayed lactogenesis stage II [10] and early frequent breastmilk expression is required to positively influence milk supply [11]. Mothers of preterm infants usually need to express milk rather than feed at the breast [12]. Beyond the neonatal intensive care unit (NICU) and medical conditions associated with prematurity, infants must transition from nasogastric (NG), cup or bottle feeds to directly feed at the breast. Other than physiological stability, there is no clear consensus on the optimal timing to commence oral feeding [13].

When infants do feed at the breast it is difficult, for both parents and healthcare professionals, to determine sufficient milk transfer. An estimated 82% of preterm infants experience some difficulty with oral feeding [14] and transitioning from nasogastric, cup or bottle feeds to feeding at the breast is complex. Oral feeding requires co-ordination of sucking, swallowing and breathing and infants must be able to generate adequate suction pressures to maintain latch and effectively remove milk from the breast. Therefore, close monitoring of breastmilk intake and growth is required to ensure optimal nutrition.

Parents recognise the importance of breastmilk for their preterm infants [15] and one of their biggest concerns both during hospital admission and post discharge is insufficient milk intake [16, 17]. However, feeding expressed breastmilk by bottle rather than latching to feed is a risk factor for early discontinuation of breastfeeding [18] as it is time consuming to both express milk and feed by bottle [15]. Moreover, those who express breastmilk to feed are more likely to report low milk supply [18].

To address these specific challenges, some neonatal units have introduced dedicated International Board Certified Lactation Consultants in NICUs and have demonstrated benefits in breastmilk provision [19]. However, it remains a challenge to support the transition to latching to feed and continued breastfeeding after discharge. In a sample of infants born at < 1750 g, Tully et al. [20] reported rates of any latch feeding at the breast were 37% while in hospital and 29.7% after discharge. By 2 months post discharge only 4.4% of mothers reported exclusive breastfeeding and 6 months after discharge no infants were exclusively breastfed [20].

Test weights have historically been used to assess intake during breastfeeding, but validity depends on rigorous protocols and precise measurements. Use of isotope labelled water to assess breastmilk intake requires multiple samples over time, but may be useful in select research settings. Clinical observation tools designed to estimate the adequacy of breastfeeds, are non-invasive and some can be used by mothers after discharge from hospital. Unfortunately, from review of the literature, no clinical observation tools have been shown to accurately reflect volumes of breastmilk intake in preterm infants [21].

## History of test weighing

Test weighing, defined as the “measurement of an infant’s breastmilk intake by weighing the infant before and after feeding without changing the napkin or otherwise altering the infant’s clothing” [22] has been used to assess milk transfer during breastfeeding for over a century [23]. Comparison of test weights with known volume feeds is documented since the 1970s [24] leading to debate in the literature. Variations in scales, weighing protocol, management of clinical equipment and interpretation of results have all contributed to this debate.

In 1979, Culley et al. [25] compared test weights to known volumes of infant formula in 115 infants showing a strong correlation (r = 0.84). At the time, test weights were frequently used in practice, but scale resolution was 5 g. Woolridge et al. [26] refined test weighing methods by using electronic scales allowing for more precise recordings and comparing feed weights to test weights, reporting correlation of 0.99. In these early studies of term infants, only correlation was reported [25, 26].

Theoretical concerns about test weight accuracy have been raised due to insensible water loss between measurements and variable density of breastmilk. Hendrickson [27] estimated insensible losses at 0.03 ml/kg/min for infants in the first year of life by performing repeated weights between feeds. Dewey et al. [28] used their own experimental measurements of insensible water loss (0.048 g/kg/minute), however this estimation remains unvalidated. Others have suggested that insensible losses within single feeds have negligible influences on test weights that take place over minutes [26, 29]. While preterm infants have increased insensible losses compared to term infants [30], this is primarily due to immature epithelial skin layer at birth [31]. Transepidermal water loss decreases as skin barriers mature with postnatal age [31] and skin barriers are widely considered comparable to term infants by 34 weeks gestation [32], when infants are typically considered clinically stable to begin oral feeding.

The density of transitional milk is approximately 1.035 g/ml and this conversion is widely used [26, 28, 33]. Other studies use 1 g/ml conversions and suggest that insensible water loss and milk density have neutralising effects [34, 35]. The conversion factor for preterm formula and fortified breastmilk may be affected by the increased specific gravity of fortified milk [36] and is relevant when comparing test weights to known volumes as most preterm infants use fortified expressed breastmilk or preterm formula when establishing oral feeding. For this reason, measuring exact weight of feeds is useful for validation purposes.

With the introduction of digital scales and dampening features negating movement effects, weights are more precise and reproducible. However even with modern scales, there is debate over the accuracy of test weights. To assess test weights in late preterm and term infants in day-to-day clinical practice, Savenije and Brand [37] performed test weights without standardised protocols. Although all weights were performed by the same investigator and the mean difference in test weight and feed volume was only 1.3 ml, the standard deviation was 7 ml with a wide 95% CI of –12.4 ml to 15 ml. Milk spilling was noted in a high proportion (21%) and, as expected, there was higher mean difference in those who had spilled milk. The authors concluded that test weights were too imprecise to be used in practice. This study highlights the need for standardised protocols when using test weights and raises concerns about using test weights outside of controlled settings.

## Test weights in preterm infants

Test weights have been evaluated in preterm infants whose precise volumes of milk intake may be clinically relevant. Meier et al. [29] compared test weights recorded on electronic and mechanical scales demonstrating superior precision of electronic scales. A population of infants born at <38 weeks, <1.8 kg, clinically stable and on full oral feeds was chosen by Haase et al. [38] to reflect infants who could potentially feed at the breast. 6.25% of test weights were outside a predetermined acceptable $\pm$ 5 g difference from actual feed volumes. The mean absolute difference was 1.97 ml with maximum difference of 10 ml and strong correlation of 0.97. Rankin et al. [39] assessed test weight validity in preterm infants with corrected gestational ages 28-36 weeks. The mean absolute difference was 2.95 ml and 85% of measurements fell within $\pm \,$ g of the actual feed weight. Of note, when a protocol concern was raised, only 71% of measurements fell within $\pm \,$ g versus 89% when no protocol deviation was noted. In addition, larger errors were noted in those with lower gestational age who weigh less and require smaller feed volumes.

For all 3 studies, investigators were blinded to known feed volumes until test weights were complete and all used a pre-defined acceptable difference of $\pm \,$ g [29, 38, 39]. Only Rankin et al. noted the type of milk used and corresponding density. Test weight agreement showed no significant difference based on feed density, but the study was not adequately powered to identify these differences [39].

## Test weighing and exclusive breastfeeding

Few studies have specifically looked at test weights and their relationship to breastfeeding success. Maastrup et al. prospectively examined a Danish cohort of preterm infants and found that test weighing during establishment of oral feeds was positively associated with achieving exclusive breastfeeding at discharge based on logistic regression analysis [40].

Test weighing is a common practice in Sweden where there are comparatively high breastfeeding rates in both term and preterm infants [41, 42, 47]. One retrospective cohort study showed that infants whose supplemental feed volumes were weaned by protocol of 5 ml/day (provided that weight gain remained adequate) had lower rates of exclusive breastfeeding compared to the test weighing cohort (OR = 2.76) [41]. The weaning group still had a 76% exclusive breastfeeding rate at discharge compared to 85% in the test weight group. In contrast, Funkquist et al. [42] performed a similar retrospective comparison of two neonatal units that employed different methods of assessing milk transfer during breastfeeds; one used test weights, the other used clinical judgement (including assessment of latch, suck and swallow) to determine the volume of supplemental milk given after each breastfeed. In infants 28-36 weeks gestational age at birth, there was no significant difference in rate of exclusive breastfeeding at discharge (88% versus 81%).

In 2004, Hurst et al. [43] analysed maternal experience of using test weights outside controlled settings with weights performed by mothers at home. This prospective randomised control trial included ex-preterm infants in the first month after discharge from hospital. Only two thirds completed the study protocol that required mothers to record all feeds, test weights, supplementary feeds, stool and urine output over a four-week period. There was no difference in partial or exclusive breastfeeding rates. Overall, only 8/31 (25%) of dyads were exclusively breastfeeding 4 weeks post discharge from hospital. Of note, infants who were small for gestational age were excluded discounting a proportion of ex-preterm infants who are at risk of undernutrition.

## Test weighing and breastfeeding duration

Both a large prospective cohort study and secondary analysis of a randomized trial have failed to identify a relationship between test weight use and breastfeeding duration [40, 43]. According to Maastrup et al.’s Danish cohort study, use of test weights was not related to breastfeeding duration which was described as adequate (defined as 4 months plus half of the time before the estimated date of delivery) versus inadequate [40]. In the study by Hurst et al. [43] examining ex-preterm infants using test weights at home, breastfeeding duration assessed via follow up interview was similar with mean 5.9$\pm$4 months in the test weight group and 6.6$\pm$3 months in the control group. Of note, only two thirds (19/31) of participants who completed the protocol were contactable for follow up.

## Concerns about test weights

While test weighing is common in many countries, practices vary and many do not routinely use test weights. Concerns around test weighing involve potential interruption of the mother baby interaction and undermining of confidence if milk transfer is low [41]. Flacking et al. [44] hypothesized that routine test weights would over medicalise breastfeeding and draw focus to measurements inaccessible to mothers post discharge from hospital. In another survey, nursing staff felt that without test weights, mothers were more focused on the experience and observation of their baby during feeds, than on weight change [41].

However, qualitative reports examining the effect of test weights have shown that test weighing in hospital had no detrimental effect on maternal confidence [45]. In mothers of ex-preterm infants using test weights at home after discharge, there was no greater anxiety or worry about amount of breastmilk received in the intervention group and those in the control group felt that having the scales would have been helpful [43].

Lastly, test weights performed without validated protocols, are subject to clinically significant errors [37] and inaccurate supplementation of feeds. Infants who receive less than required volume are at risk of poor growth and undernutrition which may result in an energy deficit and negatively impact ability to take subsequent oral feeds. Undernutrition in preterm infants has also been associated with longer term neurodevelopmental outcomes [46]. Infants who receive more supplementary volume than required are at risk of overfeeding which may exacerbate fat accretion in preterm infants and subsequent metabolic and cardiovascular risks [47]. In addition they may not display expected hunger cues and could experience slower transition to breastfeeds.

## Clinical assessment tools for breastfeeding

Alternative methods of determining breastmilk intake have been proposed and studied. Isotope labelled water has been used in breastfeeding dyads as it requires less interference immediately after feeds and does not require additional training for parents. Coward et al. [24] introduced this method using deuterium labelled water (^2^H_2_O) to measure milk transfer from mother to baby. Mothers are given a dose of ^2^H_2_O that is then delivered to infants via breastmilk transfer. Infant saliva or urine samples are subsequently measured to analyse the excretion of labelled water over time. These samples are taken at multiple time points and the intake of breastmilk is calculated based on compartmental models of distribution of body water. This method provides information about total intake over several days or weeks, but not individual feeds. When applied to preterm infants, this method is no more informative than measuring weight gain over time as it does not provide real time information to support decisions about supplemental feeds [24, 48]. For validation, estimation of breastmilk intake using isotope labelled water was compared to test weights with no significant difference between the two methods in a sample of 9 term mother-infant dyads over a 5 day period [49].

Several attempts have been made to develop clinical indicators of effective breastfeeding and milk transfer, but to date none have been validated. Meier et al. [50] demonstrated that clinical assessment of breastfeeds by an experienced observer (certified lactation educator) without a defined checklist or tool did not accurately estimate volumes of breastmilk transfer. Differences between clinical estimates and test weights appeared random with large variation. Despite this evidence, clinical observation continues in practice and is often the only tool that mothers have at their disposal when making decisions about supplemental feeding at home.

## Clinical observation tools for breastfeeding in preterm Infants

The Preterm Infants Breastfeeding Behaviour Score (PIBBS) [51], Preterm Breastfeeding Assessment Tool (PBAT) [33], and LATCH score [52] are structured clinical assessment tools that have been designed or used for evaluating breastfeeding in preterm infants. All three have been compared to test weight measurements, but none have accurately reflected volumes of breastmilk transfer.

The PIBBS was developed in collaboration with neonatal staff and mothers and was designed to identify emerging breastfeeding competence over time [51]. PIBBS has good inter rater reliability [53] but poor correlation with test weight measurements [54]. The PBAT was able to identify when no milk transfer occurred, but otherwise was not able to quantify breastmilk intake during breastfeeding [33]. Altunas et al. [55] designed a prospective study involving 33 preterm infants to compare LATCH scoring by nursing staff with test weights. LATCH scores are based on 5 components (latch, audible swallow, type of nipple, comfort, and hold) each scored 0-2 [52]. Preterm infants were assessed during oral feeds between 34 and 37 weeks corrected gestational age. The study was well blinded as a different nurse performed each measurement (pre weight, LATCH score, post weight). Although higher LATCH scores were associated with higher volumes of intake, there was significant variability of LATCH scores and the authors concluded that scores could not reliably be used to assess feed volumes.

A recent cohort study by North et al. [56] examined both LATCH and PIBBS scores in infants with low birth weight (1500–2500 g) and found higher scores at 1 week of age were associated with increased likelihood of regaining birthweight by 2 weeks, but actual feed intake was not measured. Although preterm infants represented 46% of the study cohort, mean gestational age was 37 (±2.7) weeks.

Yu et al. [57] used a Delphi panel to identify key indicators for feeding assessment of preterm infants which included rooting, latching on (duration), strength of sucking, time of sucking, longest sucking burst and swallowing. These indicators are all covered in the PIBBS assessment. The Neonatal Oral Motor Assessment Scale (NOMAS) [58] and Early Feeding Skills scale (EFS) [59] are complex assessments designed to assess sucking patterns in preterm infants and have only been tested in bottle feeding infants. NOMAS scores, do not predict feeding outcomes such as time to oral feeds [60].

A review by Pados et al. [21] concluded that no observation tool has sufficient validity and reliability to be recommended for use in clinical practice. As no other valid, practical or less intrusive methods of quantifying breastfeeding have been identified, test weights have been used as the gold standard for quantifying milk transfer during individual breastfeeds [61].

## Conclusion

Across Europe, breastfeeding rates among very low birth weight infants are 67% and 26% for any and exclusive human milk feeding at discharge [62]. When infants are fed at the breast, it is difficult to assess the volume of milk ingested. In practice, infants frequently receive a ‘top up’ volume after feeding at the breast likely undermining confidence in breastfeeding and contributing to low rates of latching to feed. Preterm infants are also at risk of over supplementation with potential detrimental metabolic outcomes or disinterest in subsequent feeds [42]. Conversely, under supplementation may result in poor weight gain, sub optimal nutrition and lack of energy to complete feeds.

The pattern of low breastfeeding rates in hospital and high attrition after discharge continues despite parental knowledge that breastmilk is important for preterm infants. Consideration must be given to interventions that can support mothers to establish and continue breastfeeding their preterm infants.

Despite structured approaches to clinical observations, none have successfully quantified milk transfer during breastfeeding in preterm infants. Test weights have demonstrated agreement with feed intake and reproducibility in controlled clinical settings. Test weights remain the standard against which clinical assessments of milk transfer have been compared in research settings and have demonstrated some association with achieving exclusive breastfeeding. However, test weights could potentially interfere with breastfeeding experience and frequent use becomes cumbersome. A pragmatic combination of clinical assessment and measurement tools may be a useful approach. Accurate measurement tools should be used to facilitate transition to direct breastfeeding while minimising consistent over or under supplementation. Ideally, these tools could be used to demonstrate milk transfer at the breast when required, without being required routinely at each feed.
